# The Sodium Iodide Symporter (NIS) as an Imaging Reporter for Gene, Viral, and Cell-based Therapies

**DOI:** 10.2174/156652312799789235

**Published:** 2012-02

**Authors:** Alan R Penheiter, Stephen J Russell, Stephanie K Carlson

**Affiliations:** 1 Department of Molecular Medicine, Mayo Clinic, Rochester, MN 55905; 2 Division of Hematology, Mayo Clinic, Rochester, MN 55905; 3 Department of Radiology, Mayo Clinic, Rochester, MN 55905

**Keywords:** Gene therapy, imaging, NIS, oncolytic virus, PET, reporter gene, SPECT, sodium iodide symporter.

## Abstract

Preclinical and clinical tomographic imaging systems increasingly are being utilized for non-invasive imaging of reporter gene products to reveal the distribution of molecular therapeutics within living subjects. Reporter gene and probe combinations can be employed to monitor vectors for gene, viral, and cell-based therapies. There are several reporter systems available; however, those employing radionuclides for positron emission tomography (PET) or singlephoton emission computed tomography (SPECT) offer the highest sensitivity and the greatest promise for deep tissue imaging in humans. Within the category of radionuclide reporters, the thyroidal sodium iodide symporter (NIS) has emerged as one of the most promising for preclinical and translational research. NIS has been incorporated into a remarkable variety of viral and non-viral vectors in which its functionality is conveniently determined by *in vitro* iodide uptake assays prior to live animal imaging. This review on the NIS reporter will focus on 1) differences between endogenous NIS and heterologously-expressed NIS, 2) qualitative or comparative use of NIS as an imaging reporter in preclinical and translational gene therapy, oncolytic viral therapy, and cell trafficking research, and 3) use of NIS as an absolute quantitative reporter.

## INTRODUCTION

Advances in preclinical and clinical tomographic imaging systems have enabled non-invasive imaging of reporter gene products to reveal the temporal and spatial biodistribution of molecular therapies in virtually any location within living subjects. Imaging of molecular therapies is critical to our understanding of patient-to-patient variability in treatment response, the development of new treatment strategies, and for patient safety monitoring. The three most common reporter gene imaging approaches use genes that encode an enzyme, receptor, or transport protein, and each of these approaches has its strengths and weaknesses [1, 2]. Ironically, one of the oldest examples of an imaging reporter gene in humans, the sodium iodide symporter (NIS), has emerged as one of the most exciting and promising reporter genes in preclinical and translational research. NIS is normally expressed on the basolateral surface of thyroid follicular cells and mediates uptake of plasma iodide (its physiologic substrate). In addition to iodide, NIS has the ability to transport several other atoms or molecules including perchlorate, perrhenate, astatide, tetrafluoroborate, and pertechnetate. 

There have been many excellent review articles on NIS published over the past 10 years [3-23]. These articles describe in great detail the molecular structure, function, and regulation of NIS, particularly as it applies to imaging and therapy of the normal and diseased thyroid gland. This review will provide a basic overview of the structure and function of NIS, but will focus on 1) differences between endogenous NIS and heterologously-expressed NIS, 2) qualitative or comparative use of NIS as an imaging reporter to monitor temporal and spatial vector distribution in preclinical and translational gene therapy, oncolytic viral therapy, and cell trafficking research, and 3) the use of NIS as a sensitive and quantitative reporter to determine the number of vector-engaged cells in a given volume of tissue.

## STRUCTURE, FUNCTION AND REGULATION OF NIS IN THE THYROID

The biosynthesis of thyroid hormones has been extensively described in medical textbooks and in the literature [5, 24, 25]. The following is a brief summary of the role of NIS in that process. NIS is an intrinsic plasma membrane glycoprotein containing 13 membrane spanning alpha helices and 3 N-linked glycosylation sites. NIS is normally located on the basolateral surface of thyroid follicular cells and co-transports two sodium ions down the electrochemical gradient and one iodide ion up the electrochemical gradient from the plasma into the cell. The process is referred to as iodide transport, and mutations in NIS are causative for congenital iodide transport defects [10, 23, 26]. The iodide transport process is driven by energy dependent transport of sodium ions through the activity of the Na^+^/K^+^-ATPase, which is also located on the basolateral membrane of the thyroid follicular cell. The main regulator of NIS expression in the thyroid is the thyroid stimulating hormone (TSH) [27, 28]. TSH stimulation of the TSH receptor initiates a G-protein-mediated signaling cascade that ultimately results in an increase in NIS mRNA and protein in thyroid follicular cells, and regulates the post-transcriptional phosphorylation and plasma membrane targeting of NIS [29-33].

## TRAPPING AND STORAGE OF IODIDE AND IODINATED MOLECULES IN THE THYROID

After iodide is transported by NIS into the cytoplasm of the follicular cell, it is either oxidized or it flows down the electrochemical gradient to the thyroid lumen (or colloid) where, under normal physiological conditions, it is oxidized and stored in the form of iodinated tyrosyl residues on thyroglobulin for eventual catabolism into thyroid hormones. The two-component vectorial transport of inorganic iodide from blood to follicular cell cytoplasm to extracellular thyroid lumen is referred to as the thyroid trap [34-36] and serves as the foundation for the timing of the clinical pertechnetate scan, generally performed 20 min post IV injection of 1-5 mCi of ^99m^Tc0_4_. Quantitative estimates indicate that under normal conditions approximately 90% of the thyroid ^99m^Tc0_4_ signal at 20 min is in the extracellular compartment [34-36]. ^99m^Tc0_4 _is a valuable imaging tool to measure this effect since it is efficiently transported by NIS but is not oxidized or incorporated into biological molecules and thus can resolve the rapid and reversible trapping activity of the thyroid from the long-term storage component. There have been many reports in the endocrinology and nuclear medicine literature describing molecular imaging techniques to distinguish iodide transport, trapping, and storage functions in the normal and pathologic thyroid tissue [37-43]. For a thorough summary, the interested reader is referred to the excellent prospective study by Reshchini *et al.* [44].

## ENDOGENOUS NONTHYROIDAL NIS BIODISTRIBUTION AND FUNCTION

NIS is also normally expressed in the salivary glands, stomach, and lactating mammary glands at levels sufficient to visualize these organs with radioiodide if imaging is performed shortly (within a few hours) after administration of radioiodide [45]. NIS in the stomach and salivary glands is thought to function in an entero-thyroid recirculation loop to prolong the retention of iodide prior to renal elimination [46], while NIS in lactating mammary glands is thought to provide a source of dietary iodide in milk [45]. In addition, NIS has been documented, using immunohistochemistry and/or reverse-transcriptase polymerase chain reaction (RT-PCR), in several other human tissues including the lachrymal glands, choroid plexus, pituitary gland, ocular ciliary body, small intestine, pancreas, adrenal gland, heart, lung, thymus, prostate, ovary, kidney tubules, placenta, testis, and rectum. With the exception of the small intestine (absorption of iodide) and renal tubules (reabsorption of iodide) the physiological role, if any, of NIS in these additional tissues remains unknown [3, 47-53]. The primary differences between NIS expression and function in nonthyroidal tissues versus its role and function in the thyroid gland are that 1) nonthyroidal NIS is not regulated by TSH; rather it appears to be constitutively expressed in most of these other tissues, 2) nonthyroidal tissues have no or minimal ability to oxidize inorganic iodide to iodine, and 3) nonthyroidal tissues do not possess the specific machinery for storage of iodine in the form of iodinated compounds. However, like the thyroid gland, other tissues which endogenously express NIS (for example, stomach and salivary glands) do so in a highly polarized fashion, where the fate of NIS-transported iodide is secretion down the electrochemical gradient to an adjacent extracellular compartment (gastric juice, saliva) opposite the pole of NIS expression [54].

## HETEROLOGOUS NIS IN TUMOR TISSUES

In contrast to the two-compartment trapping in the thyroid and the two-compartment secretion reaction in other tissues which endogenously express NIS, the majority of roles for NIS as an imaging *transgene* are for tumor cells which are not polarized. Thus, what is measured in a so-called iodide uptake assay of non-polarized cells *in vitro* and presumably what is seen on a SPECT image of a NIS-transduced xenograft is intracellular iodide. Interestingly, when directly comparing one-compartment intracellular iodide accumulation (*in vitro* assay), overexpression of NIS in a non-thyroidal cell line often results in an even higher magnitude of iodide uptake than that observed in thyroid cell lines [55]. This high level of transport activity combined with the high density of cells in many tumor types consistently provide a window of sensitivity for the detection of NIS-mediated accumulation of radioiodide or ^99m^Tc0_4_, despite the lack of a second compartment. While the majority of reports on the NIS transgene have focused on tumor cells and tumor models, a number of recent reports have explored its use as a reporter gene or surrogate gene for gene therapy and cell trafficking protocols. These normal tissue applications of NIS add an additional level of complexity to the mechanistic study of heterologously-expressed NIS, where polarized cells and second compartments could play a role in NIS-mediated imaging.

## THE BEGINNING OF NIS AS AN IMAGING REPORTER

The NIS gene was first cloned from the rat by Dai *et al.* in 1996 [56]. This was followed by cloning of human NIS later that year by Jhiang, *et al.* [57] and the cloning of mouse NIS by Pinke, *et al.* in 2001 [58]. Transient and stable NIS expression in nonthyroidal tissues and tumor xenografts stimulates significant iodide uptake *in vivo, *although to a lesser degree than *in vitro* NIS-mediated uptake, and has led to the use of NIS as an imaging reporter in numerous pre-clinical and two clinical research protocols [7, 55, 59-106]. 

Shimura, *et al.* [59] were the first to demonstrate *in vivo*, non-invasive molecular imaging with the NIS transgene. While working with FRTL cells (an untransformed, TSH dependent, “normal” thyroid epithelial cell line originating in Fisher 344 rats) [107], they noted the appearance of a cell type with altered morphology, a loss of thyroglobulin expression, and a reduction in the requirement of exogenous TSH beginning at passage 50. These altered cells were grown for an additional 40 passages in the absence of TSH giving rise to a population of cells (denoted FRTL-Tc) that did not require TSH, likely due to compensatory adrenergic responsiveness and elevated baseline cAMP. The cells; however, still responded to TSH. Also, they were no longer subject to contact inhibition, and were capable of growing as syngeneic and metastatic tumors in Fischer 344 rats. This was the first report of a malignant thyroid cell line with documented retention of the TSH receptor. Further characterization of the cell line revealed that, in addition to loss of thyroglobulin, the cells had lost the ability to accumulate iodide in an *in vitro* uptake assay [59]. This prompted the investigators to stably transfect the FRTL-Tc line with the recently cloned rat NIS gene, which restored *in vitro* iodide transport activity to a level greater than that observed in the TSH-stimulated parental FRTL cells. Tumors were then grown from these cells in Fischer 344 rats. The rats were given a tracer dose of ^125^I, and serum and tumor ^125^I concentrations were determined. A group of animals was anesthetized and serially imaged on a phosphorimaging cassette. This allowed the investigators to monitor the time course of the appearance and disappearance of radioactivity in the tumor, thyroid, and whole body. Additional rats were euthanized and absolute radioactivity levels were determined *ex vivo*. At peak tracer uptake (2 h post-injection of ^125^I) the FRTL-Tc-NIS tumors exhibited a mean of 19.3-times the serum^ 125^I concentration and a remarkable 48.3-times the control FRTL tumor concentration. Thus, if one assumes a uniform concentration of radiotracer and that each tumor cell is an independent iodide transporting entity, then one can calculate in this model that as few as 5% NIS-expressing cells will result in a tumor that appears twice as “hot” as serum. Likewise, using the same 2-fold above background as a threshold, only 2% NIS-transduced cells would be required to resolve a NIS tumor from a control tumor. These landmark studies firmly established NIS as a sensitive and quantitative imaging reporter. Mandell (1999) [60] went on to document the *in vivo* visualization of stable NIS-transduced melanoma xenografts using ^123^I scintigraphy with a clinical gamma camera. The following year, Boland *et al.* (2000) and Spitzweg *et al.* (2000) [4, 55] published imaging results with non-replicating adenoviruses encoding NIS in cervical cancer, breast cancer, and prostate cancer xenografts. This was followed by the engineering of the first replication-competent oncolytic adenovirus expressing NIS and imaging of cervical cancer xenografts and dog prostate [66]. Soon after, the first engineered replicating oncolytic RNA virus (Edmonston-strain measles virus) expressing NIS (MV-NIS) was rescued by Dingli *et al. *(2004), [108] and images of MV-NIS infected myeloma xenografts were presented. These pioneering studies paved the way for an explosion in the use of NIS as a non-invasive imaging reporter over the next decade.

## THE ROLE OF NIS AS AN IMAGING REPORTER GENE

The three main areas of preclinical and translational research to which NIS reporter imaging has been applied are viral-mediated gene therapy, oncolytic viral therapy, and cell trafficking (eg, stem cell delivery). Each of these will be discussed in further detail. The success of these research strategies depends on adequate delivery of the vector or transduced/ transfected cell to the target tissue, as well as persistent gene expression or viral infection in the target tissue. Noninvasive monitoring of gene expression or viral propagation over time in living animals is critical to facilitate the advancement of these new molecular therapies in human subjects. 

### NIS Reporter Imaging in Viral-Mediated Gene Therapy Research

The NIS reporter has been used for monitoring viral-mediated cancer gene therapy (defined here as the use of viral vectors that are not intended to lyse cells directly, but rather require expression of a therapeutic gene). In the vast majority of reports in this arena, NIS itself has been employed as the therapeutic gene with the intention of using therapeutic radioisotope concentration as the means of tumor ablation. Many of the pioneering studies in this field were instrumental in optimizing small-animal imaging methodologies that were later applied to all avenues of NIS research [4, 6, 55, 59, 64]. However, in these studies the unique reason for imaging was not to report but to “predict” the dose of a subsequently administered therapeutic isotope. As dosimetry is a fundamentally different concept than reporting (dosimetry requires the area under an imaging curve, and non-reporter background probe activity is included in the quantitative analysis) it is not the subject of this review. Interested readers, however, are referred to several recent reviews on the subject [16, 17, 109].

NIS also has been incorporated as an imaging reporter for conventional gene therapy protocols. In this context NIS can be used as a direct reporter for a gene delivery vector or to report on a second therapeutic gene. Niu *et al.* (2004) [75] investigated the use of NIS as a reporter to noninvasively image *in vivo* gene transfer and expression in lung tissue for the purposes of developing a gene therapy strategy for cystic fibrosis by potentially correcting a mutated CFTR gene and restoring its function [75]. These investigators used ^99m^TcO_4 _scintigraphy and ^124^I PET imaging to determine the location, magnitude, and duration of pulmonary gene transfer following the delivery of Ad-hNIS to the lungs of cotton rats. Lungs infected with Ad-hNIS were still visualized at 17 days post-instillation of the virus through the rat nostrils.

Another recent use of NIS reporter gene imaging has been in cardiovascular gene therapy research [83, 110-112]. Miyagawa *et al.* (2005) [110] first demonstrated the feasibility of NIS for myocardial gene expression imaging in rats using ^99m^Tc0_4_ and ^123^I scintigraphy and demonstrated a significant correlation between quantitative image analysis and *ex vivo* gamma counting of radioactivity in the NIS transfected hearts. Lee *et al.* (2005) [83] investigated the accuracy of scintigraphy for assessing myocardial gene expression in living rats using a dual-gene adenovirus that expressed both NIS and eGFP [83]. Results showed increased radioactivity uptake in a viral titer-dependent manner, and *ex vivo* analyses revealed a quantitative relationship between NIS mediated imaging and GFP expression. Rao *et al.* (2007) and Ricci *et al.* (2008) [111, 112] then went on to demonstrate the feasibility of micro-SPECT/CT imaging and quantitation of cardiac gene expression after NIS gene transfer in cardiac transplants in rats [111, 112]. Ricci *et al.* also demonstrated the high concordance between NIS reporter gene expression in the transplanted heart (measured on ^123^I scintigraphy) and soluble reporter peptide levels in serum using a bicistronic adenoviral vector expressing NIS and either human carcinoembryonic antigen or beta human chorionic gonadotropin.

### NIS Reporter Imaging in Oncolytic Viral Therapy Research

Because of their ability to selectively replicate and spread in cancer cells and their ability to amplify vector-associated transgene expression, replication-competent oncolytic viruses are being used increasingly in cancer therapy [113-118]. The ability to monitor the delivery and intratumoral propagation of oncolytic viruses is critical to understand the kinetics of oncolytic viral spread, determine the optimal viral dose for safety and maximal therapeutic effect, determine the timing of additional therapeutic strategies to enhance the oncolytic viral effect, and to improve future oncolytic viral design (targeted viruses, etc.). To achieve these goals, oncolytic viruses have been modified to express NIS.

Barton *et al.* (2003) were [66] the first to use NIS as a preclinical reporter to monitor and optimize oncolytic virus therapy. These investigators used a replication-competent adenovirus encoding a yeast cytosine deaminase (yCD)--herpes simplex virus thymidine kinase (*mut*TK_SR39_) fusion protein and a second transgene encoding NIS in mouse xenograft and dog prostate models. This same group of investigators went on to publish two additional studies to further develop and optimize this adenovirus-mediated therapy protocol in small and large animal models [94, 119] and ultimately to demonstrate the safety and feasibility of NIS and ^99m^TcO_4_ SPECT/CT as an imaging reporter system in humans [120, 121]. 

Dingli *et al.* (2004) [69] first rescued the recombinant Edmonston vaccine strain of measles virus (MV-Edm) encoding the NIS gene and demonstrated 1) the ability of MV-NIS to replicate almost as efficiently as unmodified MV-Edm, 2) human multiple myeloma tumor cells infected with MV-NIS efficiently localized radioiodide *in vitro*, and 3) MV-NIS expressing human myeloma xenografts in immunocompromised mice could be visualized *in vivo* using serial ^123^I scintigraphic imaging. Dingli *et al.* demonstrated the ability of ^124^I PET/CT to accurately image stably NIS-transfected and IV MV-NIS infected multiple myeloma xenografts [81]. Since these early experiments, other investigators have shown similar results supporting the use of MV-NIS as an imaging reporter in oncolytic viral therapy studies of ovarian cancer, pancreatic cancer, prostate cancer, and mesothelioma [88, 103, 122-124]. NIS also has been included as a reporter in other oncolytic viruses including vesicular stomatitis virus (VSVDelta51-NIS) [91], a variety of oncolytic adenoviruses [66, 92, 106, 125-127], and recently, oncolytic vaccinia virus [128].

### NIS Reporter Imaging in Cell Trafficking Research

A further application of the NIS transgene reporter system that we will discuss is its use for monitoring the delivery and fate of transduced or transfected cells with therapeutic capabilities (eg, stem cells, immune cells). Stem cell transplantation is a promising therapeutic option for patients with impaired organ function (*eg*, heart, spinal cord) due to cell death; and the use of stem cell therapy in preclinical and clinical research is expanding [16, 129-131]. Stem cells are pluripotent or omnipotent cells obtained from bone marrow and peripheral blood (among other sites in the body) that can self-renew and differentiate into specialized cells (including tissue-regenerating cells) depending on their microenvironment. Similar to the questions that arise in gene and viral therapy research, being able to determine the 1) stem cell biodistribution and homing, 2) number of stem cells that were successfully engrafted and underwent differentiation, and 3) stem cell survival time are critical to the further advancement of this research field. Thus, reporter genes are needed to help track the fate of stem cells *in vivo* and maximize the functional benefit of stem cell therapy in patients.

Thus far, cell trafficking studies have used *in vitro* cell labeling with either iron oxide nanoparticles (for use with MRI imaging) or radionuclides (for use with nuclear medicine imaging modalities) [16, 129-131]. The problem with this approach is that it is nonspecific; the imaging signal can persist even after death of the labeled cell, impairing the ability to accurately quantitate successful stem cell engraftment. The imaging reporter gene approach can overcome this limitation because the transduced cell will only provide an image signal in viable reporter gene-expressing cells. 

Stem cell therapy is a particularly hot topic in cardiovascular research because of its ability to improve cardiac function after myocardial infarction [129, 131]. Terrovitis *et al.* (2008) [129] was the first to report the use of NIS to track stem cells delivered to the rat heart with SPECT and PET imaging. The investigators transduced rat cardiac-derived stem cells (rCDCs) using lentiviral vectors and injected them intramyocardially (up to 4 million NIS+ rCDCs) immediately after left anterior descending coronary artery ligation. Cell viability and proliferation was not affected by NIS expression, and ^99m^TcO_4_ SPECT and ^124^I PET imaging demonstrated NIS-mediated uptake of radiotracer in the areas of myocardial perfusion deficit. Higuchi *et al.* (2009) [131] used NIS-mediated ^124^I PET imaging to successfully monitor the delivery and survival of endothelial progenitor cells (EPCs) after transplantation into the rat heart (*ex vivo* myocardial tissue sections confirmed that the ^124^I signal was linearly related to the number of EPCs present in the heart). They also showed that pretreatment of the rat with a combination of Atorvastatin and VEGF led to a prolongation of early survival of the transplanted EPCs.

NIS can also be used as a reporter gene for monitoring immune cell trafficking *in vivo*. Seo *et al.* (2010) [132] published the first study using NIS reporter gene imaging to monitor macrophage migration towards inflamed tissue [132]. These investigators used an immortalized macrophage cell line genetically engineered to express NIS and GFP (RAW264.7/hNIS-GFP) and small animal PET imaging with ^18^F-FDG and ^124^I to image an area of chemically-induced inflammation in a nude mouse thigh that was created by the intramuscular injection of turpentine. The rate of cell proliferation, cytokine production, and phagocytic activity were not affected by the insertion of the NIS and GFP dual reporter transgenes. PET images obtained with both ^18^F-FDG and ^124^I showed a similar “doughnut-shaped” area of uptake at the inflammation/injection site in the rat thigh. The migration of macrophages to this inflamed site was further confirmed by *ex vivo* IHC staining. 

## THE ADVANTAGES OF MICRO-SPECT AND MICRO-PET FOR NIS IMAGING IN SMALL ANIMALS

NIS-mediated transport activity *in vivo* allows the use of standard nuclear medicine imaging modalities including planar gamma-camera (scintigraphy) and single photon emission computed-tomography (SPECT) using ^99m^TC, ^125^I, and ^123^I, and positron emission tomography (PET) using ^124^I and ^18^F-tetrafluoroborate. Prior to the development of dedicated small animal imaging systems most preclinical imaging employed large human clinical systems or laboratory instruments designed for other purposes, such as a phosphorimager. Over the past 10 years, significant advances in instrumentation and image reconstruction software have led to the development and commercialization of multiple dedicated small animal SPECT and PET systems [133-143]. 

Currently, planar scintigraphy is more widely available, is less expensive than cross-sectional imaging with SPECT or PET, and has been the most widely used imaging modality for preclinical NIS research. The planar gamma camera remains a useful tool because it enables the determination of the whole-body biodistribution of an injected radionuclide. In fact several small animals can be imaged simultaneously due to the large field of view afforded by the gamma camera. However, the planar gamma camera has considerable limitations due to its low resolution and because it represents a 3-D distribution of radioactivity in a 2-D display [144, 145]. This results in superimposition of overlying radioactivity, difficulty with accurate localization of abnormalities, and errors in radiotracer quantitation [146]. In addition to these general problems, there are also potential challenges with the NIS reporter for *in vivo* planar scintigraphy with radionuclide-based techniques because of high radiotracer uptake in the stomach that may result in a strong signal on the 2-D scintigraphic images. This may lead to decreased spatial resolution and inaccurate monitoring of NIS expression in adjacent abdominal organs such as the liver, spleen, and pancreas. 

Cross-sectional imaging techniques such as SPECT and PET are needed to improve 3-D spatial resolution and separate the overlapping regions of radioiodine uptake *in vivo*. Combined multi-modality scanners have recently been developed that allow fusion of functional (SPECT or PET) data with high-resolution anatomical (CT) data to provide a more accurate localization and quantitative estimate of radioactivity in live animals. A tri-modal small-animal PET/SPECT/ CT system was also recently introduced [141]. 

Although PET is more often used than SPECT for clinical applications because of its higher sensitivity, resolution, and quantitative advantages for deep-tissue imaging, this is not the case for small animal imaging. There are several excellent recent review articles comparing the advantages and disadvantages of micro-SPECT and micro-PET imaging in preclinical research [141, 143, 147-149]. Briefly, micro-SPECT and micro-PET both use similar detector systems and record the emission of photons *in vivo* to document the location of an administered radiotracer. The type of decay that each of the system detects, however, is different (single gamma photon emission for SPECT versus two photon emission from positron annihilation for PET) and therefore requires the use of different radionuclides. 

Two of the biggest differences between small animal SPECT and PET are with regard to image sensitivity and resolution. SPECT imaging requires the use of a lead collimator to detect and convey information about the origin and direction of the gamma photon for representation in a 3-dimensional image format. Because lead collimators reject the majority of emitted gamma photons (>99%), the sensitivity of SPECT is approximately 2 orders of magnitude lower than PET and therefore requires higher doses of administered radiotracer and/or increased imaging time [143, 147, 148]. Cheng *et al.* (Cheng 2010) compared two commercially available small animal SPECT and PET imaging systems with regard to image sensitivity and resolution by imaging a mouse flank xenograft using ^99m^Tc0_4_ and ^18^F-labeled tumor-specific nanoparticles. Their results demonstrated 15-fold higher sensitivity with PET for detection of tumor radioactivity. The trade-off, however, is that SPECT can employ multiple pinhole collimators to provide higher *spatial resolution* than PET (down to < 0.5 mm for micro-SPECT versus 1 to 2 mm for micro-PET) [143, 148]. Marsee *et al.* [74] were the first to image NIS expression with micro-SPECT/CT and were able to detect NIS-expressing orthotopic lung tumors as small 3 mm with ^125^I micro-SPECT using pinhole collimation. Newer generation micro-SPECT systems offer sub-millimeter image resolution with increased sensitivity using a detector with a larger surface area and increased field-of-view [138, 140]. Targeted pinhole micro-SPECT can also be used to increase SPECT imaging sensitivity [142] within a small field of view.

Several researchers have used ^124^I PET imaging to document *in vivo* NIS expression [65, 71, 75, 81, 128, 150]. Groot-Wassink, *et al.* showed a strong correlation (r = 0.9581) between PET imaging quantitation (%ID/g) and *ex vivo* analysis of ^124^I hepatic uptake in mice systemically administered a NIS-expressing adenovirus [71]. Dingli, *et al.* [81] used a clinical PET/CT scanner to document and quantitate NIS-mediated ^124^I uptake in stable NIS-expressing (25% ID) and MV-NIS infected (7.1%) mouse myeloma xenografts. The drawbacks associated with use of ^124^I are that it is not readily available, its production is complex, and the tissue penetration prior to annihilation of the high energy positrons from ^124^I decay (maximum positron range of >6 mm) severely limits the spatial resolution in a small animal setting [151]. A recent breakthrough in NIS research is the development of a novel PET probe ^18^F-tetrafluoroborate ([^18^F]TFB) [152, 153]. Although not yet widely used, this may replace the less sensitive and lower resolution ^124^I for PET imaging of NIS. 

In addition to image sensitivity and resolution, there are several other factors to consider with regard to choosing an imaging modality for NIS. Advantages of SPECT compared to PET for small animal studies are that SPECT is 1) less expensive, 2) able to image multiple probes labeled with different radiotracers of different energies at one time allowing the visualization of more than one molecular event, 3) ability to use radiotracers that are readily available at on-site pharmacies and that do not require an on-site cyclotron for production. However, both micro-SPECT and micro-PET can provide similar highly accurate *in vivo* quantitation of radioactivity and have been thoroughly validated by direct comparison with ex-vivo analyses [71, 84, 122, 136, 143].

## RELATIONSHIP BETWEEN IMAGING ACTIVITY AND FRACTION OF NIS-EXPRESSING CELLS

Ultimately the result of quantitative imaging can be viewed as a concentration (some measure of atoms or activity *per unit of mass or volume*). In molecular imaging this is often referred to as absolute quantitation. The term “absolute quantitation” helps to distinguish it from other forms of image quantitation that are relative to internal or external controls in either a spatial or temporal manner. From the concentration value obtained from imaging, with appropriate validation experiments, it is then possible to calculate the fraction or number of NIS-expressing cells within a given volume. It is important to note here that thyroid quantitation is fundamentally different in this regard. The results of quantitative thyroid image analysis are usually presented as % injected dose with no denominator. The reasons for this are 1) the ability of the thyroid to compete with all other forms of radioiodide tracer *clearance *in the body is an important physiological property of the gland, and 2) when concentration is required (such as in dosimetry calculations), a standard organ mass is often used. 

In theory, both CT and SPECT (or PET) are absolutely quantitative because imaging instruments are calibrated with standards so the reconstructed CT dimensions and SPECT or PET voxel (a volumetric or three-dimensional pixel) intensity exactly match the true dimensions and true radionuclide concentration within the voxel. In humans, however, attenuation of photons and Compton scatter result in considerable loss of photons reaching the detector in comparison to instrument calibration standards where the surrounding medium is air.

In mice, with medium energy photons such as those from ^99m^Tc decay (140 keV) or positron annihilation (511 keV), attenuation and scatter losses are minimal. However, a different impediment to accurate quantitation is encountered—the resolution of small animal micro-SPECT/CT or micro-PET/CT devices is much lower than human SPECT/CT or PET/CT relative to anatomical volume. Thus, large corrections are required for partial volume effects, where the majority of counts are projected (displayed) outside of the volume from which they emanated. With a spherical tumor of uniform activity, this partial volume effect can be corrected for based on standard curves generated from imaging spherical phantoms of known dimension and activity Fig. (**1**) [84]; however, for different shaped tumors and non-uniformly distributed signals, deviations from the standards are often too large to provide accurate quantitative data. 

Additionally, at the CT settings typically employed for small animal imaging, [84, 103] even subcutaneous tumors are only weakly resolved from underlying skeletal muscle, surrounding fluid, and overlying skin. These difficulties in small animal SPECT/CT and PET/CT have resulted in a general bias where tumors for quantitative imaging studies are often much larger than those employed for therapy studies in the same model. In a recent paper [122], we attempted to address these issues by using 1) 1-mm pinhole SPECT collimation, 2) SPECT-based volume of interest (VOI) analysis, 3) empirical tumor-based CT optimization for accurate measurement of *in vivo* subcutaneous tumor dimensions, and 4) tumor volumes that are more typical of established tumor models for therapy (0.1 to 0.6 cm^3^). We directly compared the *in vivo* imaging measurements to *ex vivo* measurements immediately following imaging. Fig. (**2**) Using a threshold of 1.5-fold above control tumor uptake (background), we calculated 2.7% MV-NIS-infected BxPC-3 tumor cells were required for detection within this model. Additionally, by measuring the volume of BxPC-3 tumor cells and the tumor cell/stroma ratio, we can calculate that with the imaging settings employed (2.2 mm voxel size), approximately 2 x 10^5^ infected tumors cells are required to reliably resolve a zone of infection from background in this model Fig. (**3**). We have applied the same general techniques to orthotopic pancreatic tumors stably expressing NIS Fig. (**4**), and to document multiple sites of MV-NIS injection and infection within a single flank tumor Fig. (**3**). 

A number of other reports have demonstrated the ability of the NIS transgene to serve as an absolute quantitative reporter for gene therapy vectors. Groot-Wassink *et al.* (2004) [71] performed a study with a non-replicating adenoviral vector expressing a CMV promoter-driven NIS (Ad-hNIS). They administered increasing doses of Ad-hNIS (5 x 10^6^ to 2.5 x 10^9^ pfu) to C57/B6 mice via tail vein injection. To validate the quantitative reporter aspects of the transgene, an ex-vivo comparison of NIS-mediated ^124^I accumulation and NIS RNA (as a surrogate for infected cells) was performed. The results indicated a nearly perfect correlation between ^124^I quantitation and NIS RNA in both the threshold dose to establish a significant infection and linearity with increasing dose above the threshold. Subsequent IHC analysis of mouse liver sections enabled an estimate of the relationship between ^124^I concentration, quantitative RNA analysis, and percent of cells infected with virus. Further experiments were performed to confirm the *in vivo* quantitative analysis with ^124^I PET. A nearly perfect linear correlation was confirmed between volume of interest image analysis and *ex vivo* measurements of radiotracer uptake. In this example, the liver is quite large relative to the resolution of the imaging system and a uniform distribution of IV administered Ad-hNIS within the liver can be assumed. Therefore, the conversion of imaging “activity” to a meaningful measure of absolute concentration (% ID/g) is straightforward. In a similar study, Lee and Kim *et al.* (2005) [83] assessed infection in living rats following intramyocardial injection of an adenovirus expressing NIS and eGFP [83]. As in the Groot-Wassink study, a threshold dose of Ad-GFP-NIS was required to establish a quantifiable infection. Beyond that, however, there was a very good correlation between % ID/g (following administration of ^123^I) and the measurement of quantitative fluorescence for GFP content in the transduced cardiac tissue.

A fundamentally different form of quantitative reporter use of NIS was tested in a study by Vadysirisack *et al.* (2006) [154]. The authors attempted to determine the utility of NIS as a gene/promoter reporter. They used quantitative immunoblotting, RNA analysis, and a tetracycline-inducible promoter to drive a gradient of NIS expression. This enabled a direct comparison between NIS mRNA levels, total NIS, surface NIS, and iodide transport activity in a synchronized manner. They observed in several different cell types a level of NIS surface protein above which additional steady iodide transport activity did not occur. This non-linear relationship between NIS mRNA, NIS protein, and NIS activity, indicates that NIS is probably not an ideal candidate reporter for a gene/promoter assay. 

Conditionally-replicating adenovirus (CRAds) may represent the most difficult challenge to the concept of NIS reporter quantitation of transduction. With CRAds there may be a clear virion per cell (gene dose) response at early time points or if the cells are minimally permissive to amplification. While in other cells, a region of low MOI infection combined with substantial amplification could result in a similar imaging signal as a high MOI infection. In order to deal with these practical issues, Barton *et al.*, [66, 119, 121] introduced the concept of gene expression volume (GEV, with units of cm^3^) and the gene expression unit (GE, which equals the GEV times the ratio of imaging intensity in the GEV to that of blood). For example, a region of interest signal of 1 cm^3^ with a mean calculated probe concentration of 3 times that of the blood would equal 3 GE units. These units could then serve as reference values for optimization of intratumoral injections where the combined volume of NIS activity and magnitude of NIS activity should reflect the total tissue dose of drug produced from a prodrug convertase encoded in the same virus. In a phase 1 clinical trial extension, Barton *et al.* 2011 [121] used a replication-competent adenovirus encoding a yeast cytosine deaminase (yCD)--herpes simplex virus thymidine kinase (*mut*TK_SR39_) fusion protein and a second transgene encoding NIS (Ad5-yCD/ *mut*TK_SR39_*rep*-hNIS). They directly injected 5 x 10^12^ Ad5-yCD/*mut*TK_SR39_*rep*-hNIS viral particles divided into twelve injection sites into the prostate of 6 patients with localized prostate cancer. The patients were imaged serially with SPECT/CT after intravenous injection of ^99m^Tc0_4_ beginning one day after administration of the virus. Quantitative SPECT/CT image analysis enabled the determination of 1) the volume of Ad5-yCD/*mut*TK_SR39_*rep*-hNIS infected prostate, 2) the concentration of ^99m^Tc within each GEV and it’s relation to adjacent organs and blood, 3) the % of prostate infected, and the 4) the rate of disappearance of infection from the prostate. 

## RADIONUCLIDE BASED REPORTER/PROBE SYSTEMS

Radionuclide-based reporter imaging techniques with scintigraphy, SPECT, or PET offer the highest sensitivity for detecting low levels of reporter gene expression (picomolar sensitivity) and appear the most suited for translation to the clinic at this time [1, 2, 155, 156]. Optical imaging techniques (with bioluminescent enzyme/substrate systems or fluorescent proteins) are useful in small animals, but are limited in humans due to the depth of tissue penetration (generally < 2cm) [157]. Magnetic resonance imaging with specific contrast agents offers high spatial resolution [158-160] but is less sensitive than radionuclide techniques and is limited by the high concentration of contrast material required to provide sufficient image contrast [161]. 

In addition to NIS, several other radionuclide based reporter systems have been utilized for preclinical and translation studies. Table **1** contains a list of these reporter proteins, along with some of the commonly employed SPECT and PET probes for each reporter. Radiological (physical) half-life, positron yield, maximum positron energy for PET isotopes, and the principle ‘imageable” gamma photon for SPECT isotopes are given for each radionuclide. Herpes simplex virus-1 thymidine kinase (TK) and an optimized mutant (*sr39tk), *which has higher affinity for imaging probes and lower affinity for thymidine, can be imaged with radiolabeled acycloguanosines or 2’-fluoro nucleoside analogs of thymidine. The Dopamine D2 receptor (DR2) and a second generation receptor (R80A), which retains ligand binding function but does mediate internal signaling, can be imaged with ^11^C, ^18^F, or ^123^I-labeled D2 antagonists. Likewise, a variety of somatostatin analogs are available for either PET or SPECT imaging of the somatostatin receptor type 2 (SSRT2). The norepinephrine transporter (NET) can be imaged with either ^123^I or ^124^I MIBG (a norepinephrine analog). Radionuclide probes for NIS are either radioactive isotopes of iodide (^123^I, ^124^I, ^125^I), or molecules with a size and charge density similar to iodide, such as ^99m^Tc0_4_ or [^18^F]-TFB.

## NIS VERSUS OTHER SPECT/PET REPORTERS

As can be seen in Table **1**, all of the commonly employed radionuclide reporters can be imaged with either PET or SPECT probes. Thus, the decision to choose one reporter or another can be made largely upon biological considerations rather than on the basis of instrumentation. Some biological considerations when choosing a reporter/probe system are (1) endogenous receptor activity, background localization of probe, location of target tissue in relation to regions of high probe concentration, and the timing and potential competition of endogenous activity and background in relation to the desired reporter signal. In this regard, NIS would not be desirable for a reporter in the stomach or bladder. However, the low background of NIS probe activity in liver, kidney, or skeletal muscle for example might be ideal for NIS imaging. (2) For conventional gene therapy approaches, it is desirable to use a self-protein as a reporter to minimize the possibility of immune-mediated destruction of cells encoding the reporter. An advantage of NIS is this regard is that in addition to being a self-protein, NIS-encoding constructs from several species including mouse, rat, human, and dog (Stephen J. Russell, unpublished), have been developed and tested in animals. (3) Also, for conventional gene therapy, it is important to consider biological activity of the ectopically expressed reporter and its effect on cell physiology (either constitutive activity or through the action of endogenous substrates). These issues have been addressed for HSV-1 TK and D2R with the engineering of vectors encoding the *sr39tk* mutant and R80A mutant, respectively [155, 162]. Theoretically, constitutive activity might be a concern for NIS. However, long term culturing of stable NIS expressing cells lines, as well as long term expression of NIS in normal animal tissue, for example, in the NIS transgenic heart mouse [163], would seem to indicate that most cells are quite able to tolerate a background level of NIS activity. (4) A final biological consideration is the size of the reporter gene—the vector must be able to accommodate the insert. In this respect, NIS is the largest of the reporters, which could limit its applications in some viruses with strict limits on insert size. 

If these biological considerations above are met, the primary advantage of NIS is that it can be used with inexpensive and readily available ^99m^TcO_4_. ^99m^TcO_4_ costs only a few dollars per dose and can be eluted daily from an in-house generator. No labeling reactions are required, and the imaging characteristics (140 keV gamma) are ideal and standardized for quantitative SPECT/CT imaging in both small animal and human instruments. A disadvantage of NIS versus some other commonly employed reporters is that the only approved PET isotope for NIS imaging is ^124^I. The high energy positrons released from ^124^I decay have a long range in tissue prior to annihilation, which limits resolution, and the yield of positrons from ^124^I is quite low relative to several other PET isotopes [164-166]. It remains to be seen if [^18^F]-TFB will be approved for human use. However, if approved, [^18^F]-TFB should provide the ideal combination of sensitivity (four ^18^F atoms per molecule) and resolution for PET imaging of the NIS reporter.

## CONCLUSIONS

As indicated throughout this review, the desirable attributes of NIS are its quantitative nature, its remarkable flexibility of iodide transport activity in many cells types using viral and non-viral platforms, and the ease in which the activity is observed *in vivo*. The main hurdle to the use of NIS transgene and all other reporter-based technology is the sensitivity (the concentration of probe within the target region versus that in the plasma or neighboring region). Sensitivity is of particular concern for oncolytic virotherapy research using replicating viruses where it is desirable to detect lower levels of infection that may cause unwanted side effects, and for situations where the goal is to obtain “proof of concept” information in a model that may not be a “best case scenario”. The hope is that with continued work in basic research to improve sensitivity, validation in relevant animal models, and ultimately translation to human clinical trials, we will see a time when the concept of imaging reporter-guided gene therapy, viral therapy, and cell-based therapy becomes a standard of care for diseases which today are untreatable. 

Additionally there is hope that in the future reporter gene imaging will facilitate more than simply accurate reporting on the spatial and temporal pattern of vectors. It is conceivable that the results of reporter imaging will both serve as a guide for confirmatory biopsies and enable clinicians to make rational decisions about additional or complementary therapies on a patient-by-patient basis 

## Figures and Tables

**Fig. (1):**
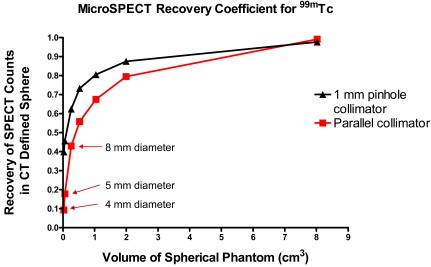
A spherical phantom study to determine the partial volume losses on a Gamma-Medica (Northbridge, CA) X-SPECT System. Images for the ^99m^TcO_4_ phantom series were obtained with a high-sensitivity parallel-hole collimator and a 1-mm pinhole collimator. The fraction of counts displayed within CT defined spheres is plotted as a function of sphere volume.

**Fig. (2):**
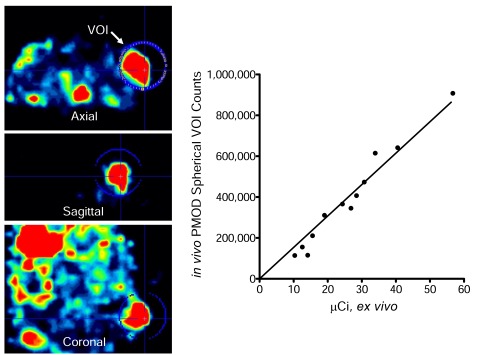
Pinhole micro-SPECT/CT image analysis versus *ex vivo* quantitation of BxPC-3 tumors 3 days post-infection with an oncolytic measles virus encoding NIS. A single spherical volume of interest (VOI) was drawn around each tumor and the activity determined with PMOD imaging software. Immediately following imaging, tumors were excised and counted in a dose calibrator.

**Fig. (3):**
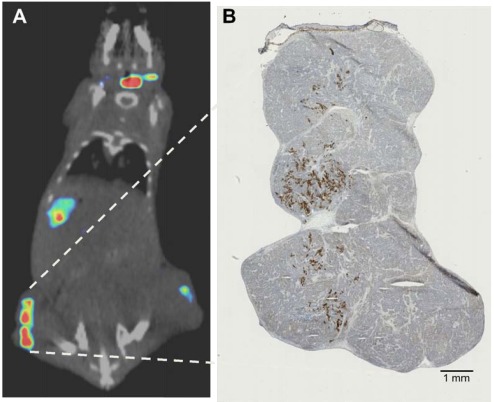
Micro-SPECT/CT image and corresponding immunohistochemistry analysis of early infection with multiple intratumoral injections of MV-NIS. A BxPC-3 xenograft was injected in three locations with a total dose of 3.5 x 10^6^ MV-NIS. On day 4 post-injection, the animal was imaged with pinhole micro-SPECT/CT. (**A**) The tumor was removed, aligned with the micro-SPECT/CT image, processed by IHC for measles N (brown staining), and counterstained with hematoxylin. (**B**) An excellent spatial correlation is seen between the live animal micro- SPECT/CT image and the three small zones of intratumoral infection.

**Fig. (4):**
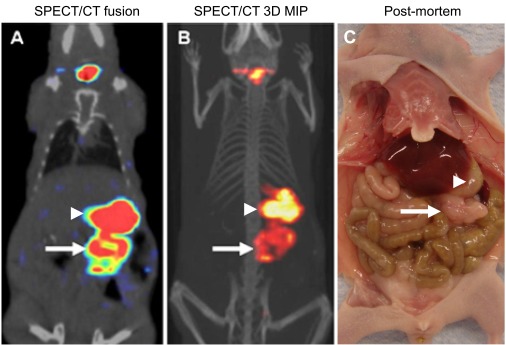
Micro-SPECT/CT imaging of an orthotopic transplantation model of pancreatic cancer. BxPC-3-NIS xenograft fragments were transplanted to the pancreas of donor mice. Twenty days after transplantation, animals were injected with 1 mCi ^99m^Tc0_4_ and imaged 1 h later. (**A**) Coronal fusion micro-SPECT/CT slice through the center of the pancreatic tumor (arrow) adjacent to the endogenous stomach activity (arrowhead). Thyroid uptake is also seen on the image. (**B**) The same imaging data set is displayed as a maximum intensity projection (MIP) generated from threshold-adjusted axial slices. (**C**) Post-mortem analysis reveals the size and location of the pancreatic tumor (arrow) relative to adjacent stomach (arrowhead) and other organs.

**Table 1.** Heterologous Reporter/Probe Imaging Strategies

Reporter	Probe	Modality	Half-life	Positron (yield), Energy	References

Dopamine D2 Receptor	[^11^C]raclopride	PET	20 min	β^+^ (100%), 0.96 MeV	[162, 167-171]
(R80A)	3-(2´-[^18^F]fluoroethyl)-spiperone	PET	110 min	β^+^ (97%), 0.635 MeV	
443 aa	[^123^I]iodobenzamine	SPECT	13.3 h	γ, 159 keV	

Herpes Simplex Virus-1 Thymidine Kinase (sr39tk)	9-[4-[^18^F]fluoro-3-(hydroxymethyl)butyl] guanine ([^18^F]FHBG)	PET	110 min	β^+^ (97%), 0.635 MeV	[77, 155, 172-180]
	1-(2-deoxy-2-[^18^F]fluoro-d-arabinofuranosyl)-5-iodouracil ([^18^F]FIAU)	PET	110 min	β^+^ (97%), 0.635 MeV	
376 aa	1-(2-deoxy-2-fluoro-d-arabinofuranosyl)-5-[^124^I]iodouracil([^124^I]FIAU)	PET	4.2 days	β^+^ (25%), 2.13 MeV	
	[^125^I]FIAU	SPECT	59 days	γ, 35 keV	
	[^18^F]-2´-Fluoro-2´deoxy-1h-D-arabionofuranosyl-5-ethyl-uracil ([^18^F]FEAU)	PET	110 min	β^+^ (97%), 0.635 MeV	

Somatostatin Receptor Type 2	^111^In-octreotide	SPECT	2.8 days	γ, 171, 245 keV	[181-186]
	^99m^Tc-octreotide	SPECT	6 h	γ, 144 keV	
369 aa	^68^Ga-dotatate	PET	68 min	β^+^ (90%), 1.90 MeV	
	^94m^Tc-demotate-1	PET	53 min	β^+^ (72%), 2.47 MeV	

Norepinephrine Transporter	meta[^123^I]iodobenzyl- guanidine ([^123^I]MIBG)	SPECT	13.3 h	γ, 159 keV	[187-190]
617 aa	meta[^124^I]iodobenzyl- guanidine ([^124^I]MIBG)	PET	4.2 days	β^+^ (25%), 2.13 MeV	

Sodium Iodide Symporter	^99m^Tc0_4_	SPECT	6 h	γ, 140 keV	[55, 59, 60, 62, 65, 152, 153, 191]
	^123^I	SPECT	13.3 h	γ, 159 keV	
643 aa	^125^I	SPECT	59 days	γ, 35 keV	
	^124^I	PET	4.2 days	β^+^ (25%) 2.13 MeV	
	[^18^F]tetrafluoroborate	PET	110 min	β^+^ (97%), 0.635 MeV	

Legend: R80A refers to Arg to Ala substitution at residue 80; *sr39tk* is a thymidine kinase mutant with increased activity against acycloguanosines; γ, gamma photon, β^+^, positron.
